# *Actinomyces odontolyticus* bacteraemia associated with cervical and mediastinal abscesses in an immunocompetent patient: First reported case in Qatar

**DOI:** 10.1016/j.nmni.2022.100956

**Published:** 2022-01-17

**Authors:** Almurtada Razok, Maisa Ali, Loai Aker, Hisham Ziglam

**Affiliations:** 1) Department of Internal Medicine, Hamad Medical Corporation, P.O 3050, Doha, Qatar; 2) Department of Infectious Diseases, Hamad Medical Corporation, P.O 3050, Doha, Qatar; 3) Department of Clinical Radiology, Hamad Medical Corporation, P.O 3050, Doha, Qatar

**Keywords:** *Actinomyces odontolyticus*, Bacteraemia, Descending mediastinitis, Mediastinoscopy, Parapharyngeal abscess

## Abstract

The *Actinomyces* bacteria are associated with cervicothoracic disease in immunocompromised patients; however, *Actinomyces odontolyticus* cervical infection with extensive spread to the mediastinum in a previously healthy patient was not reported before in Qatar. The patient underwent drainage of collections in synchrony with intravenous antibiotics and recovered with an excellent outcome.

## Highlights

• *A. odontolyticus* was historically linked with dental and periodontal disease.
• *A. odontolyticus* can cause infections in immunocompetent and compromised patients.
• Cervicofacial infections can spread to the mediastinum and surrounding structures.
• Combined medical and surgical treatment is essential for a successful recovery.

## Introduction

The *Actinomyces* species are part of the *Actinomyceataceae* family that was identified in the 19th century. Initially thought to be a type of fungi, their appearance in human disease was first described in 1857 [1,2]. They are gram positive, anaerobic bacteria that contribute to the flora of the gastrointestinal and genitourinary tracts [3]. These bacteria can cause infections of the central nervous, respiratory, and gastrointestinal systems and can therefore present in a variety of clinical pictures.

## Case report

**Fig. 1:**
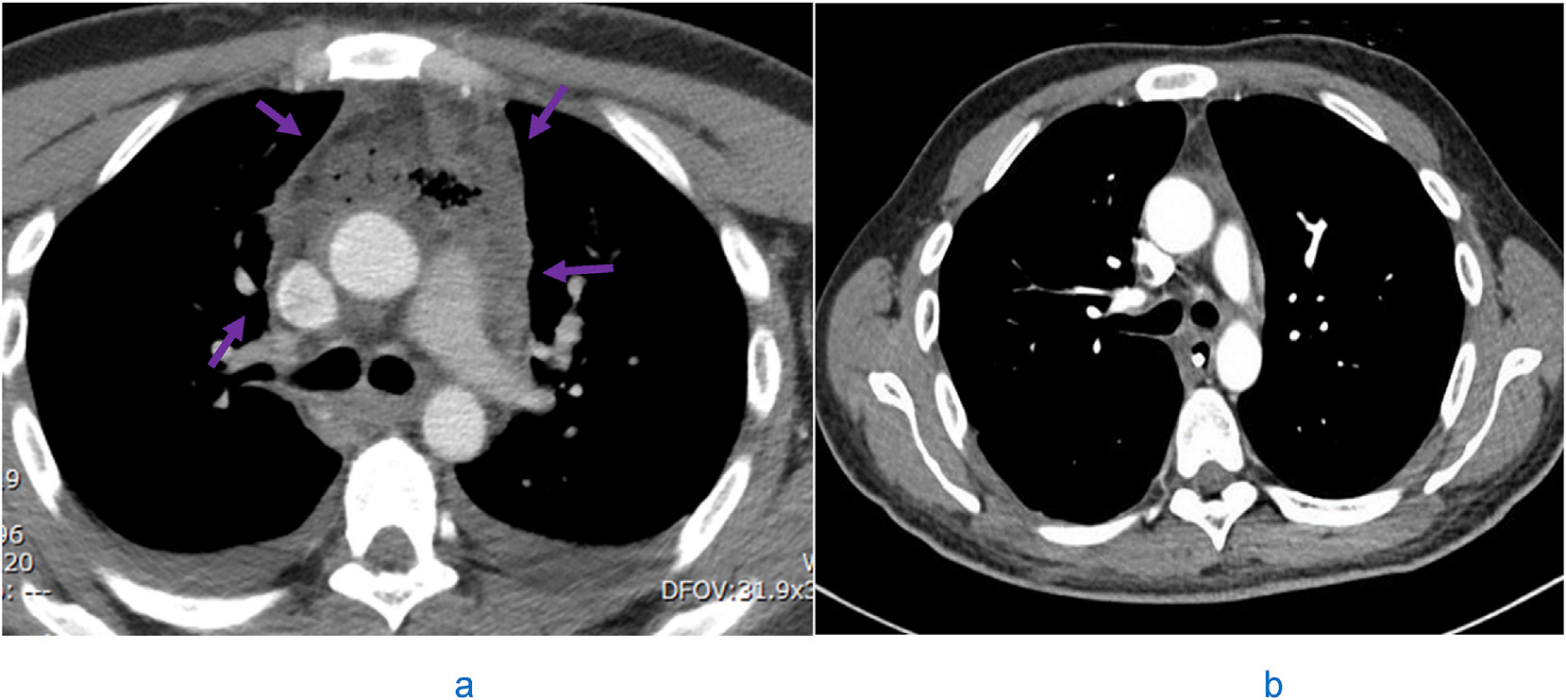
a: Axial section of contrast-enhanced computed tomography scan of the neck and thorax at the level of the tracheal bifurcation shows descending necrotizing mediastinitis complicated by abscess formation in the superior and anterior mediastinum (purple arrows). b: Axial section of contrast-enhanced computed tomography scan of the thorax shows total resolution of the mediastinal abscesses (performed three months post-operatively).

A 32-year-old gentleman with no previous medical history, presented with fever, right-sided facial swelling and trismus. He was a non-smoker and non-alcoholic. He underwent extraction of tooth 48 seven days prior for the purpose of discomfort relief. There were no signs of odontitis or periodontitis at the time of the procedure and the procedure was performed in compliance with the appropriate sterility measures. Upon presentation to the hospital, the patient's temperature was 38.2 C and other vital signs were normal. Examination revealed swelling in the right submental area. Laboratory results revealed a white blood cell count of 22 × 10^3^/uL (Reference range 4-10 × 10^3^/uL) and C-reactive protein of more than 500 mg/L (Reference range 0-5 mg/L). His HBA1C was 5.2% (Desirable less than 5.7%). Computed tomography (CT) of the neck and chest revealed submental and parapharyngeal collections. There was also descending necrotizing mediastinitis complicated by abscess formation (Fig. 1a).

Piperacillin/Tazobactam was started. He underwent drainage of the submental and parapharyngeal collections in addition to mediastinoscopy and drainage of mediastinal abscesses. Blood cultures were positive for *A. odontolyticus*. The isolate was cultured anaerobically in blood agar enriched with 5% CO2 after 48 hours of incubation in a temperature of 37 C. The microorganism was presumptively identified by Maldi and confirmed with Phoenix techniques. Further confirmation using DNA restriction analysis was carried out. In addition, review of the isolate after a prolonged incubation period of two weeks was consistent with the initial identification. Cultures from mediastinal abscesses were also positive for *A. odontolyticus*. The isolates were pan-sensitive. His antibiotic was changed to intravenous Clindamycin on day four post-admission. Repeated blood cultures were negative for growth, and his post-operative course was uneventful. He recovered successfully with no major sequelae and was discharged 30 days' post-admission after receiving antibiotics for four weeks. Repeat CT scan three months post-operatively revealed complete resolution of all collections (Fig. 1b). Looking for possible immunodeficiency which may have not been identified before, the patient underwent multiple serologic testing two weeks post-discharge. These included serum immunoglobulin levels (IgA, IgG, IgM and IgE) which were in the reference range and fourth generation Human Immunodeficiency Virus (HIV) combined antigen/antibody testing which was negative. In addition, flowcytometric oxidative burst assay using nitroblue tetrazolium (NBT) ruled out neutrophil dysfunction.

## Discussion

*A. odontolyticus* was first isolated in 1958 from patients who had extensive dental and periodontal disease including caries [4]. Manifestations of infection by *A. odontolyticus* include thoracic, abdominal, pelvic and central nervous system disease. In the four decades following its isolation, more than 20 cases of invasive infections were reported in multiple geographic locations including the United States and Europe. Majority of the patients were males and the mean age at diagnosis was 50 years [5].

Thoracopulmonary infections due to *A. odontolyticus* were observed before. The first case of mediastinitis was reported in an immunocompromised patient following a heart-lung transplant and required combined medical and surgical treatment with intravenous penicillin and sternal debridement, respectively. Unfortunately, the patient did not survive the infection [6]. Other intrathoracic infections include pneumonia, empyema and chest wall abscess, with most cases reported in immunocompromised patients. Pneumonia was reported in alcoholic, chronic lung disease and transplant patients [7,8]. Empyema and pulmonary abscess were reported in patients who had underlying pulmonary disease and rheumatoid arthritis [9]. A case of empyema due to *A. odontolyticus* was also reported in an alcoholic patient who had history of pulmonary tuberculosis and aspergilloma which were treated with pneumonectomy. He underwent drainage, received antimicrobial agents, and responded to treatment [10].

A review of the epidemiology of *A. odontolyticus* in the state of Qatar between the years 2016 and 2020 revealed 15 cases of bacteraemia. Nine of the patients were females (60%). Mean age at diagnosis was 45 years. 11 patients (73%) had comorbid conditions, including hypertension and diabetes mellitus. 12 patients (80%) had fever on presentation. Two patients (13%) presented with upper respiratory symptoms, two with urinary tract infection and two with lower respiratory complaints, with the remaining patients presenting with constitutional symptoms. Three patients (20%) had orofacial abnormalities in the form of dental caries. Most of the patients (60%) received B-lactam antibiotics. One patient passed away while the other 14 recovered uneventfully with a case fatality rate of 6.6%.

In summary, we describe the case of a 32-year-old immunocompetent gentleman who presented with fever and facial swelling seven days after dental extraction and was found to have *A. odontolyticus* bacteremia associated with cervical collections and descending necrotizing mediastinitis. The patient underwent surgical drainage of the collections in synchrony with intravenous antibiotics and successfully recovered with an excellent clinical outcome.

We believe that this case is unique due to multiple reasons. First, case reports of such an extensive cervical and mediastinal involvement in a previously healthy patient following a simple, uncomplicated dental extraction were not published. Secondly, the patient fully recovered despite a relatively short period of antibiotics which was four weeks only, as the usual recommended duration in cases with extensive cervical and thoracic involvement is 3-6 months, even when combined with surgical drainage [11]. In addition, this case highlights the importance of combined medical and surgical management in patients with *A. odontolyticus* cervical and thoracic collections and how it helps to minimise the duration of antibiotics.

## Ethics approval

An approval from Hamad Medical Corporation, Medical Research Center (MRC) was obtained prior to submission of this manuscript. Manuscript number MRC-04-21-064.

## Consent for publication

Written informed consent was obtained from the patient for publication of this case report and the accompanying images. A copy of the written consent is available for review by the Editor-in-Chief of this journal on request.

## Availability of data and materials

The datasets used and/or analyzed during the current study are available from the corresponding author on reasonable request.

## Funding statement

This article did not receive any specific grant from funding agencies in the public, commercial, or not-for-profit sectors.

## Author contribution statement

AR performed literature review and wrote the original draft of the manuscript. MA and HZ supervised the writing process and revised the manuscript. LA performed literature review and provided the radiology images with their corresponding interpretation. All authors approved the final version for submission.

## Transparency declaration

None to be declared.
